# Lightweight green composite of bacterial cellulose/tungsten oxide nanowires for attenuation of gamma radiation

**DOI:** 10.1038/s41598-026-58375-4

**Published:** 2026-07-07

**Authors:** Ola E. A. Al-Hagar, Aya M. Matloob, Deyaa Abol-Fotouh, Ahmed H. M. Solieman

**Affiliations:** 1 Plant Research Department, Nuclear Research Center, Egyptian Atomic Energy Authority (EAEA), Cairo, 13759 Egypt; 2 Refining Department, Egyptian Petroleum Research Institute (EPRI), Nasr City, Cairo 11727 Egypt; 3 Advanced Technology and New Materials Research Institute (ATNMRI), City of Scientific Research & Technological Applications (SRTA-City), Alexandria, 21934 Egypt; 4 Experimental Nuclear Physics Department, Nuclear Research Center, Egyptian Atomic Energy Authority (EAEA), Cairo, 13759 Egypt; 5 Cyclotron Facility, Egyptian Atomic Energy Authority (EAEA), Cairo, 13759 Egypt

**Keywords:** Bacterial cellulose, Tungsten oxide nanowires, Gamma ray attenuation, Narrow beam setup, FTIR, Physics, Biomaterials, Composites

## Abstract

Polymeric composites are recently recommended for substituting the traditional radiation shielding non-eco-friendly lead-based materials. Sustainable polymer cellulose has been studied for shielding against multiple types of radiation, but the bacterial cellulose (BC) has not received the same attention for this application although of its extraordinary physico-chemical properties such as the high crystallinity and the entangled nanofibrous structure which secure high robustness, high reactive surface area, porosity, in addition to its green footprints. Herein, we examined the potentiality of BC composites in the attenuation of gamma radiation by combining it with multiple proportions of tungsten oxide nanowires (WO_3_ NWs). The synthesized WO_3_ NWs underwent TEM, Zeta potential, and Zeta sizing analyses, while other structural characterization has been conducted on the BC/WO_3_ NWs composites including FTIR, XRD, SEM, EDX, contact angle, TGA, where these investigations declared the compositing between the BC and WO_3_ NWs. WO_3_ showed their diversified sized nanowires shape under the SEM and the TEM detector, with negative surface charge (-22 mV) revealed by *Zeta* potential. EDX analysis of the BC/WO_3_ NWs elucidated the inclusion of tungsten metal, while the contact angle analysis indicated decreased hydrophilicity degree for the BC/WO_3_ NWs comparing to the pristine BC. The TGA analysis pinpointed that the BC/WO_3_ NWs composite showed thermostability till T = 247 °C, before the structure start collapsing. Afterward, four BC/WO_3_ NWs constructs of multiple weight percentages were tested for their gamma radiation shielding, where the examined composite exhibited an attenuation capability directly proportional to the WO_3_ NWs loading whenever the photon energy was below about 250 keV. Above this photon energy, no obvious shielding impact for the BC/WO_3_ NWs composite was observed, regardless of the dopant concentration. This suggests the potential of the BC/WO_3_ NWs in attenuation of gamma radiation at low energy range and proposes reinforcing the composite composition to extend the scale of its shielding capacity for higher energy range.

## Introduction

Studying novel materials for shielding gamma radiation grabs ascending concern of the scientists globally, as gamma ray incidence has escalated in different domains such as nuclear plants, healthcare field, food radiance, research domains, and goods` sterilization units^1^. Moreover, conventional radiation shielding materials, such as lead-based materials, are no longer compatible with the environmental standards or even meeting the safety demands of most applications. Global restrictions on lead-based materials necessitate their replacement with lightweight, cost-effective, flexible, and non-toxic alternatives^2,3^.

Recently, polymer composites have been widely recommended as efficient radiation shielding materials. Compared to pure metallic shields, polymeric composites are commercially accessible, present lower environmental impact, and offer advanced functional attributes such as optical tunability, lightweight character, and mechanical flexibility. Additionally, polymer-based materials show chemical and thermal stability, and produce fewer secondary photons upon reacting with the radiation^4,5^.

To attain high potentiality of radiation attenuation, the selection of the appropriate polymeric matrix and the relevant dopant (as a filler) is a critical factor, as the radiation interaction is totally relying on the chemical composition of the shielding material^6^. On the other hand, multiple applications are demanding shielding materials with high conformation capability^1^. One successful strategy is attained by downsizing the shielding dopants into the nano-scale and maximizing their proportions packed in the polymeric matrices^6,7^. Additionally, it was widely reported that the filler in the nano-scale showed higher attenuation coefficients compared to the corresponding micro-sized form^8–10^. Tungsten (W) and W-based composites are distinguished examples for these shielding fillers owing to their nontoxicity, high density, and high atomic number^11^. Chang and his group have investigated the attenuation capacity against gamma radiation of tungsten/epoxy composite, where they reported the direct proportionality between the attenuation capacity and the tungsten loading^12^. Likewise, the poly (hydroxyethyl methacrylate-co-styrene)/tungsten (VI) oxide composite has attained its best attenuation value at the higher tungsten loading (wt. 50%) at the adopted three photon energies (662, 1173, 1332 keV)^13^. Interestingly, Wang et al. have compared the flexible composites of the pure W/Bi and W/Bi oxides regarding their attenuation of gamma and (X) rays, explaining how the oxide forms of these elements exhibit relatively weaker attenuation capability because of the oxygen content, while, on the other hand, the W/Bi oxides showed better dispersion and homogeneity in the final composite form^14^.

Cellulose-based composites have garnered significant attention as potential shielding materials against various types of radiation. Their renewable origin, biodegradability, and biocompatibility make them attractive alternatives to synthetic polymers, especially in environmentally conscious applications. Unlike conventional plastics, cellulose offers a green footprint with minimal ecological impact throughout its lifecycle. This sustainability is coupled with promising functional properties, particularly when engineered into advanced composite forms. Researchers have explored its use in electromagnetic shielding, X-ray attenuation, and even neutron moderation, depending on the fillers and structural modifications. Such versatility positions cellulose composites as a leading candidate in the development of next-generation, eco-friendly radiation protection systems^15,16^. For instance, Maluangnont and his coworkers have tested a composite of cellulose/titanate nanosheets for shielding gamma radiation^17^. Additionally, high viscosity carboxymethyl cellulose (HVCMC) have been combined with poly N-vinyl pyrrolidone (PVP) and polyethylene glycol (PEG) through polymer blinding route, where the resulting composite was examined for its gamma radiation attenuation^1^. Moreover, X-ray attenuation potentiality of the transparent cellulose/BaSO_4_ composite was investigated by Jiang et al.^18^ A composite of cellulose acetate/CdO-ZnO was evaluated *versus*the concrete and gypsum regarding the X-ray shielding^19^.

Bacterial cellulose (BC) is a typical cellulose produced in a fully pure form by certain microorganisms as a tri-dimensional web of nanofibers of 10–100 nm diameter. BC is typically produced through entirely water-based process, and the final product is naturally non-toxic and eco-friendly^20,21^. Due to the specific biological conditions under which BC is synthesized, the resulting material demonstrates remarkable mechanical strength, characterized by its exceptional tensile properties. Furthermore, the biosynthetic process promotes a highly ordered molecular arrangement, giving BC its elevated degree of crystallinity. In addition, the unique microbial production pathway generates a network of interconnected nanofibers, which endows the material with significant porosity, thereby enhancing its potential for diverse applications^22^. Additionally, the polymer exhibits an abundance of hydroxyl group (OH) that facilitates the chemical modification, while it shows high resistance for dissolving by most organic solvents or harsh acid/base treatment^23^.

Although BC has been widely studied for protection against electromagnetic waves and X-ray, the studies for investigating shielding against gamma radiation still scarce. Uniquely, Kołodziejczyk and his team reported the promising gamma attenuation capacity of the wet films BC and BC supplied with metals (Fe, K, & Mxenes)^24^.

While BC shares the environmentally friendly and sustainable nature of other biopolymers—such as alginate, gelatin, chitosan, and conventional plant-derived cellulose (PC)—its unique physical and structural characteristics render it significantly superior. Unlike PC, which typically requires extensive extraction and purification processes to obtain a usable form, BC is biosynthesized by microbes in an inherently pure state, eliminating the need for costly and energy-intensive refinement steps^25,26^.

Moreover, BC is naturally produced in the form of nanofibers, with diameters approximately 500 times thinner than those of PC. This nanoscale morphology is achieved without the need for additional chemical treatments or mechanical processing, which are often necessary to convert PC into nanocellulose^27,28^. In contrast, alginate exerts poor mechanical strength and limited chemical stability, while chitosan has been widely reported to exhibit inadequate mechanical performance. Gelatin, although biocompatible, is highly susceptible for enzymes, shows thermolability and lacks structural integrity under elevated temperatures^29,30^.

These comparative advantages position BC as a highly promising polymeric candidate for advanced applications, particularly in the development of lightweight radio-shielding materials. Its combination of nanoscale architecture, mechanical robustness, and intrinsic purity makes it exceptionally well-suited for functional composites requiring both structural performance and environmental sustainability.

In the current study, we examined the potentiality of BC/WO_3_ NWs composite to attenuate gamma radiation using “narrow beam” setup. The BC/WO_3_ NWs which fabricated of different weight percents were tested adopting radiation sources Ba-133 [ε: 100%], and Eu-152 [ε: 72.08%; β-: 27.92%].

To the best of our knowledge, this study is the first to investigate the gamma radiation shielding capabilities of a novel lightweight nanocomposite composed of sustainable bacterial cellulose (BC) and highly attenuating tungsten oxide. This composite material is synthesized *via* an environmentally friendly, water-based process, offering a promising alternative to traditional radiation shielding materials that are typically heavy, non-sustainable, and impractical for many applications. By integrating the mechanical flexibility and biodegradability of BC with the superior shielding efficiency of tungsten oxide, this approach aims to address the growing demand for green, lightweight, and effective radioprotective solutions.

## Methodology

### Biosynthesis of bacterial cellulose (BC)

Bacterial cellulose (BC) was produced using the strain *Komagataeibacter hansenii* KO28-05D, which was originally isolated from the Egyptian environment using the facilities of the Microbiology Laboratory, Plant Research Department, Nuclear Research Center, Egyptian Atomic Energy Authority (EAEA). To enhance BC production, the strain was exposed to two low doses of gamma radiation (0.5 kGy) *via*a Cobalt-60 gamma irradiator (MC20, Russia) housed within the Cyclotron Project at EAEA, aiming to induce beneficial mutations. Further methodological details can be found in our earlier publication^31,32^.

To produce BC films, sterile volume of 90 mL of the Hestrin-Schramm (HS) medium in 500 mL Pyrex beaker was inoculated by 10 mL of *K. hansenii* KO28-05D culture, before the beaker sealed by aluminum paper and incubated at 30 °C in the dark for 7 days. Afterward, the films were collected and washed by boiling d-H_2_O then hot 0.1 N NaOH, twice for each; 15 min. each time. Next, the films were rinsed extensively by d-H_2_O till the neutral pH was reinstated.

Consequently, five BC films were collected and immersed in 50 mL of d-H_2_O, where they were homogenized by a hand blender for 10 min. The resulting aqueous suspension of BC macrofibers was stored at 4 °C until further examinations.

### Synthesis of tungsten oxide nanowires (WO_3_ NWs)

Tungsten oxide nanowires (WO_3_ NWs) were simply synthesized by the acidic precipitation method. First, 6.59 g of the sodium tungstate (Na_2_WO_4_) salt was dissolved in (200 mL) d-H_2_O and then with continuous stirring (600 rpm), 10 mL of hydrochloric acid (HCL) were added dropwise to the sodium tungstate solution. The mixed solution was kept under stirring for 5 h, after which the precipitates were allowed to settle for 24 h at room temperature. The precipitate was filtered using a Whatman filter paper and washed many times by d-H_2_O until pH reached 7 during the filtration process^33^. Then, the washed precipitate was dried at 100 °C in an oven for 1 h. After drying, the precipitates were calcined in muffle furnace at 500 °C for 4 h.

### Synthesis of BC/WO_3_ NWs composite

The direct blending of certain proportions of the suspension of never-dried BC macrofibres and the WO_3_ NWs powder was all-water-based approach selected to fabricate the composite. In brief, certain weights of the WO_3_ NWs powder were dispersed in equal aliquots of the BC suspension before stirring for 6 h at 50 rpm using magnetic bar. Afterward, the blend was poured in a glass beaker, before it moved to an oven of 60 °C for 24 h. The dried BC/WO_3_ NWs pellets were either utilized for the characterization plan, or compressed later-on for examining their attenuation potential, where the polymer/nanowires proportions were indicated in the corresponding section below.

### Characterization of the materials

For the characterization scheme, BC/WO_3_ NWs composite of (wt. 10%) nanowires load was prepared. FTIR (ALPHA II Compact FTIR Spectrometer – Bruker- Germany) at spectra range 4000–400 cm^− 1^ with 2 cm^− 1^ resolution for 16 scans per measurement. X-ray diffraction of the samples was analyzed using (labX XRD-6100, Shimadzu, Japan). The utilized CuKα radiation wavelength was (λ = 1.54 Å), generated at a voltage of 40 kV and a filament emission of 30 mA. The samples were scanned at 2θ range of 5°– 80° at a scan speed of 0.5° min^− 1^. Scanning electron microscope (SEM) imaging and Energy Dispersive X-ray Spectroscopy (EDX) were conducted by (Zeiss, Germany) at a voltage of 25 kV after sputtering samples with gold nanoparticles for 1 min. The nanomorphology of the synthesized WO_3_ NWs was investigated using transmission electron microscopy (TEM - Jeol 2100 plus). Dynamic light scattering (DLS) and Zeta-potential inspection of the tungsten sample were scrutinized by (Nano ZS Nanoseries Zetasizer -Malvern Instruments, UK), where the data was extracted at 25 ℃ through putting an aliquot of 300 µL of aqueous solution of the sample in a quartz cuvette (10 mm path length). Contact angle was measured using (Attension Theta lite, Biolin Scientific). Thermogravimetric analysis (TGA) was conducted in air using a TGA–DSC/DTA analyzer (Discovery SDT 650, TA Instruments, USA), with the temperature ramping from room temperature up to 800 °C at a heating rate of 10 °C/min.

### Molding of BC/WO_3_ NWs composite

To fabricate the BC/WO_3_ NWs blends: 5, 13, 80, and 250 mg of WO_3_ NWs powder were dispersed in four aliquots of BC slurry, where the four blends were dried at 70 °C for 6 h. Afterwards, the dry BC/WO_3_ NWs forms were compressed into a disc of diameter 13 mm using 7-ton hydraulic press machine, where the final WO_3_ NWs (wt%) for each BC/WO_3_ NWs disc was estimated *via* the Eq.

1 $$\:({\mathrm{wt}}.\:\% )\:{\text{WO3 = }}\:\frac{{{\mathrm{Weight}}\:{\mathrm{of}}\:{\mathrm{WO}}_{{\mathrm{3}}} }}{{{\mathrm{Total}}\:{\mathrm{sample}}\:{\mathrm{weight}}\:({\mathrm{Cellulose}}\:{{ + }}\:{\mathrm{WO}}_{{\mathrm{3}}} )}}{{ \times 100}}$$

### Measurement of attenuation coefficient

The photon measurement system consists of gamma spectrometer equipped with HPGe detector (BSI, Baltic Scientific Instruments) with resolution at 1.33 keV equals 1.8 keV and relative efficiency 40%. The above detector is coupled with a data acquisition system and digital multi-channel analyzer (MCA527 - GBS Elektronik GmbH). The spectra were analyzed for peak identification, count rate detection, and statistical uncertainty estimation using Genie™ 2000 software (Mirion Technologies, Inc). Table 1 summarizes the decay data and used gamma lines of the used isotopes for the current measurements.

**Table 1:** Decay data of the radioactive sources used for measurement of mass attenuation coefficient^34^.

Source	Half-life & Decay mode	Gamma Energy	Gamma emission intensity
		(keV)	(%)
Ba-133	(10.520y)	53.16	2.14
	ε: 100%	79.61	2.65
		80.99	32.9
		160.61	0.63
		276.39	7.16
		302.85	18.34
		356.01	62.05
		383.84	8.94
Eu-152	(13.517y)	39.52	21
	ε: 72.08%	40.11	37.7
		45.29	3.75
		45.41	7.26
		46.57	2.4
		121.78	28.53
		244.69	7.55
		443.96	2.82
		867.38	4.23
		964.05	14.51
		1085.83	10.11
		1112.07	13.67
		1212.94	1.41
		1408.01	20.87
Eu-152	(13.517y)	344.27	26.59
	β−: 27.92%	411.11	2.23
		778.9	12.93

During gamma spectra analysis, the two gamma lines 39.52 keV and 40.11 keV of Eu-152 cannot be separated within the resolution limit of the detector due to the detector’s limited energy resolution, which cannot distinguish between gamma lines separated by only 0.59 keV. At low energies, peak broadening and noise further prevent resolving these closely spaced emissions, causing them to appear as a single peak. Therefore, they treated as a single peak of average energy 40 keV. Additionally, the three gamma lines 45.29 keV, 45.41 keV and 46.57 keV of Eu-152 cannot be separated. Thus, these lines were treated as a single peak of average energy 45.5 keV.

Likewise, the two gamma lines 79.61 keV and 80.99 keV of Ba-133 cannot be separated within the resolution limit of the detector, thus they treated as a single peak of average energy 81 keV. This summation of the physical gamma-lines does not affect the accuracy of resulting attenuation coefficient measurements as the samples attenuate the incident photons regardless of their physical origin. Although this summation gives rise to a better statistical uncertainty of the measured beam-intensity due to the higher total count for the sum peak, nevertheless, it implies that the reported results would be averaged over a wider energy range.

The data of 45.5 keV gamma line was de-convoluted using Genie2000 gamma analysis program to resolve its partial interference with the 46.53 keV gamma of Pb-210 decay from natural U-238 series in the background radiation.

The gamma spectral data were collected for enough periods to obtain statistical uncertainty around 1% (except the 53.2 keV and 160.6 keV gamma lines of Ba-133 whose peak-area uncertainty reaches up to ~ 5%).

For Eu-152 isotope (point source of activity ~ 1 µCi), the spectrum data collection period was 8.2 h, where the results were averaged over the 3 measurements. The results of one measurement for 25 h were found to be nearly same as the average over three measurements of 8.2 h each. For Ba-133 isotope (point source of activity ~ 1 µCi), the spectrum data collection period was 46 h.

The attenuation coefficient was measured using the “narrow beam” setup. The collimated beam was obtained by using rectangular lead block of 10 cm thickness and 11 mm aperture diameter, where the thickness of samples was chosen be around half of the mean free path to avoid scatter buildup^35,36^.

Assuming ideal parallel narrow photon beam and absence of scatter radiation buildup, then photon attenuation through the passage into a sample shielding block obeys the exponential attenuation law:

2 $$\:I={I}_{0}\:{e}^{-\mu\:\chi\:}$$

where ***µ*** is linear attenuation coefficient of the shielding material, ***x*** is the sample thickness, ***I***_***o***_ is the incident photon beam intensity, and ***I*** is the attenuated beam intensity.

The mass-attenuation coefficient (µ_m_) has been calculated according to the relation:

3 $$\:{\mu\:}_{m}=\:\frac{\mu\:}{\rho\:}=\:\frac{1}{\rho\:\chi\:}\:\mathrm{ln}\left(\:\frac{{I}_{0}}{I}\right)\:$$

where ***ρ*** is the density of the shielding material.

The uncertainty in measurement of the mass attenuation coefficient (σµ_m_) could be derived from Eq. (3) by partial differentiation with respect to all variables (i.e. *ρ*, *x*, *I* and *I*_*o*_) and application of the general equation for the propagation of uncertainty. Therefore:

4 $$\frac{{\sigma _{{\mu _{m} }} }}{{\mu _{m} }} = \sqrt {\left( {\frac{{\sigma _{x} }}{x}} \right)^{2} + \left( {\frac{{\sigma _{\rho } }}{\rho }} \right)^{2} + \left( {Ln\left( {\frac{{I_{o} }}{I}} \right)} \right)^{{ - 2}} \mathop {}\limits^{{}} \left( {\left( {\frac{{\sigma _{I} }}{I}} \right)^{2} + \mathop {}\limits^{{}} \left( {\frac{{\sigma _{{Io}} }}{{I_{o} }}} \right)^{2} } \right)}$$

where $\sigma _{x} ,\mathop {}\limits^{{}} \sigma _{\rho } ,\mathop {}\limits^{{}} \sigma _{I} ,\mathop {}\limits^{{}} \sigma _{{I_{o} }}$ are the uncertainties in the sample thickness, density, attenuated beam count, and incident beam count, respectively.

## Results and discussion

### Characterization of the synthesized composite

The application of BC in attenuation of ionizing radiation is still uncovered, where it is presumed to endow the shielding materials with higher physical properties comparing to many other polymers including other cellulose derivatives^19,37^.

**Fig. 1:**
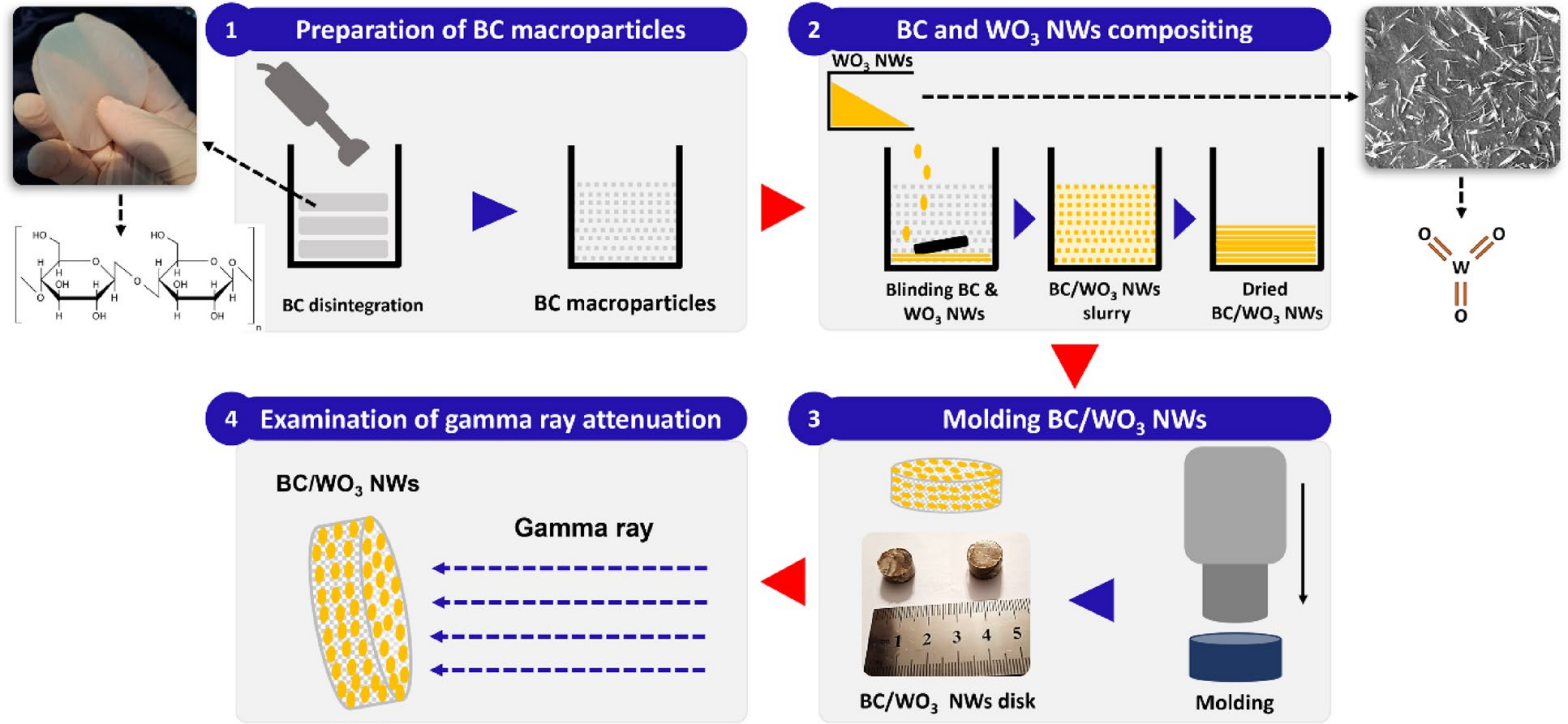
Schematic diagram indicating the main four phases for the production and examination of the BC/WO_3_ nano composite in gamma ray shielding.

The schematic diagram in Fig. 1 explains the phases of all-water preparation of BC/WO_3_ NWs, including the direct blending of the BC macro fibers and WO_3_ NWs to produce the dried pellets, before applying the high pressure to generate high dense BC/WO_3_ NWs discs.

Lightweight gamma radiation shielding materials are essential in applications where reducing mass improves performance, such as aerospace systems, portable medical devices, and wearable protection^38,39^. They enable safer and more efficient designs in space missions, where mass strongly affects launch cost and feasibility^38^. In medical imaging and radiotherapy, lightweight shields improve mobility and ergonomics compared to conventional heavy materials such as lead^40^. They also support flexible and customizable shielding solutions in industrial and nuclear environments through polymer-based composites^41,42^.

Table 2 illustrates that the bulk density of BC and BC/WO₃ nanowires is significantly lower than that of conventional shielding metals and their composites. While the density of BC is comparable to that of common polymers such as polycarbonate (PC) and polyethylene terephthalate (PET), the reduced environmental impact of this natural polymer either for production or its residues underscore the advantages of employing BC as a sustainable alternative.

**Table 2:** A list of the most prominent gamma attenuation materials according to their densities comparing to BC and BC/WO_3_ NWs utilized in the current study.

Material	Type	Density (g/cm³)	Notes	References
Bacterial cellulose (BC)	Natural polymer	1.45 ± 0.01	High crystallinity and hydrogen bonding; promising eco-friendly matrix for shielding composites	This work
BC/WO_3_ NWs	Composite	1.56–1.69 (depends on loading)	Provide a balance between density (weight) and the gamma-ray shielding efficiency	
Tungsten (W)	Metal	~ 19.3	Extremely high density and Z; excellent gamma attenuation, alternative to lead	Kim et al.^43^
Lead (Pb)	Metal	~ 11.34	Standard shielding material due to high density and attenuation coefficient	Kirshenbaum et al.^44^
Polycarbonate (PC)	Synthetic polymer	~ 1.2	Transparent shielding applications	Prajzler et al.^45^
Polyethylene Terephthalate (PET)	Synthetic polymer	1.33–1.45 (depends on crystallinity)	Poor shielding alone but improved with fillers at low energy < 200 keV	Dhaka et al.^46,47^
Polytetrafluoroethylene (PTFE)	Synthetic polymer	~ 2.2	Lightweight; poor shielding alone but improved with fillers	Marshall et al.^48^
Tungsten-filled epoxy composite	W-polymer composite	~ 7–11 (depends on loading)	high attenuation due to tungsten’s high Z and density	Kim et al.^43^
Bismuth oxide/Tungsten oxide -polytetrafluoroethylene (PTFE), polyethylene (PE), and polyetherimide (PEI) composites	Polymer composites	~ 2–6 (depends on loading)	Lead-free shielding with tunable density and attenuation	Marshall et al.^48^
Heavy concrete	Composite (cement-based)	~ 2.3–3.5	Widely used in nuclear facilities; density depends on aggregates	Subaar et al.^49^

**Fig. 2:**
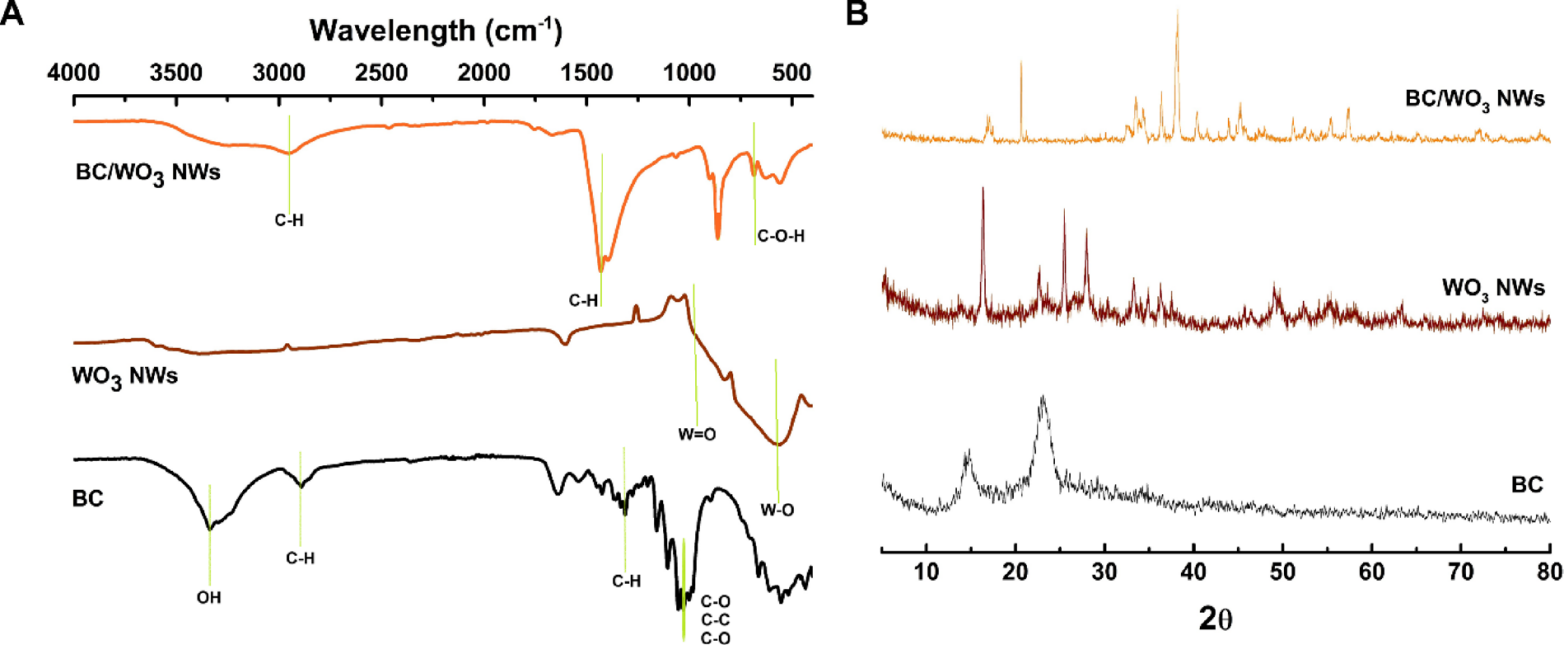
(**A**) FTIR patterns of the BC, WO_3_ NWs, and BC/WO_3_ NWs samples; (**B**) XRD of the BC, WO_3_ NWs, and BC/WO_3_ NWs samples.

In order to determine the exact composition of the samples, FTIR measurement was implemented (Fig. 2A). The pattern of the pristine BC sample was almost in complete correspondence with our previous inspection^50^. Briefly, the peak projected at 3340 cm^− 1^ is assigned to hydroxyl group stretching, while the peak at 2892 cm^− 1^ is attributed to C-H stretching. The peak at 1376 cm^− 1^ expresses the C-H bending, while the consortium 1002–1029 cm^− 1^ is attributed to C-O at C3; C-C stretching; and C-O at C6.

The monoclinic WO_3_ NWs shows a large band with characteristic frequency vibrations in the range 400–1000 cm^− 1^^51^. The band at 982 cm^− 1^ is observed in the range of (Metal=Oxygen) vibrations, which is characteristic for stretching vibrations of W = O bond. The band at 620 cm^− 1^ is attributed to W–O stretching vibrations, while the band at 714 cm^− 1^ is assigned to the W-O-W bridging modes of the WO_6_ (octahedral) corner sharing species. The peak at 1400 cm^− 1^was an unusual attribution of O-H stretching and blending vibration^52^. In addition, from this spectrum it can be proved that the structure of WO_3_ NWs does not retain any amount of adsorbed water, which is reflected by the absence of the broad band around 3380 cm^− 1^ characteristic of H_2_O stretching vibration. Meanwhile, the most evident regarding the BC/WO_3_ NWs IR pattern is the diminished band at 3349 cm^− 1^ assigned to the hydroxyl group, suggesting formation of hydrogen bonding between the OH of the BC and the oxygen of the compositing WO_3_. Moreover, the peak at 1385 cm^− 1^ may be attributed for the C-H group of the BC fraction, while the peak at 665 cm^− 1^ is assigned for the C-O-H out of plan bending or the W–O vibrational modes of the WO₃ NWs.

On the other hand, a band centered at ~ 800 cm⁻¹ can be noticed in the FTIR spectrum of the BC/WO₃ NWs composite material. This band is associated with the W–O–W bridging modes of the WO_3_units. This is a typical band found in the spectrum of tungsten oxide materials. By contrasting the spectrum of BC/WO₃ NWs composite with that of WO₃ NWs, the band centered around ~ 800 cm⁻¹ of the composite appears slightly broadened yet remains distinctly defined. This can be associated with the interface interaction between the WO₃ NWs material and the hydroxyl groups of the BC matrix, which mainly relies the hydrogen bond interaction between the surface oxygen atoms of the WO₃ material and the abundant hydroxyl groups of the BC nanofibers^11^.

Al-Sulami et al. elucidated that the compositing of WO_3_nanostructures probably enhances the IR absorbance of the composite, which may be attributed to multiple contributing factors, including the intrinsic absorption properties of WO₃, enhanced light scattering induced by the dispersed nanoparticles, and a possible increase in charge carrier concentration within the composite^53^. The following XRD results regarding crystallographic construction may come in accordance with these FTIR outcomes.

The X-ray diffractograms are shown in Fig. 2B. BC diffraction exhibited the ordinary pattern of cellulose Iα, where the triple peaks at 14.7°, 16.7°, and 22.4° which assigned to the 100, 010, and 110 planes of the Iα allomorph. The diffraction peaks (2θ = 16.2°, 22.6°, 25.5°, 28.09°, 34°, 34.9°, 38°, 49°, 52°, 56° and 62°) are assigned to the reflections associated with the tungsten oxide (WO_3_) phase, a monoclinic crystal system (JCPDS data file 01–072-1465)^54^.

The XRD pattern of the BC/WO₃ NWs composite is dominated by the characteristic diffraction peaks of the monoclinic WO₃ phase. In contrast, the diffraction signals of BC appear significantly suppressed, primarily due to the high crystallinity and strong intensity of the WO₃ nanowires, as well as substantial peak overlap—particularly between the most intense BC peak (Iα, 110 planes at ~ 22.4°) and a major WO₃ peak (020 plane at ~ 22.6°). Subtle shifts in peak positions and variations in relative intensities, compared to the pure WO₃ pattern, are indicative of microstrain and environmental alterations within the crystallites. These changes are likely attributed to physical confinement and interfacial stress exerted by the BC nanofiber matrix during composite formation and drying, which may induce lattice distortion in the embedded nanowires.

**Fig. 3:**
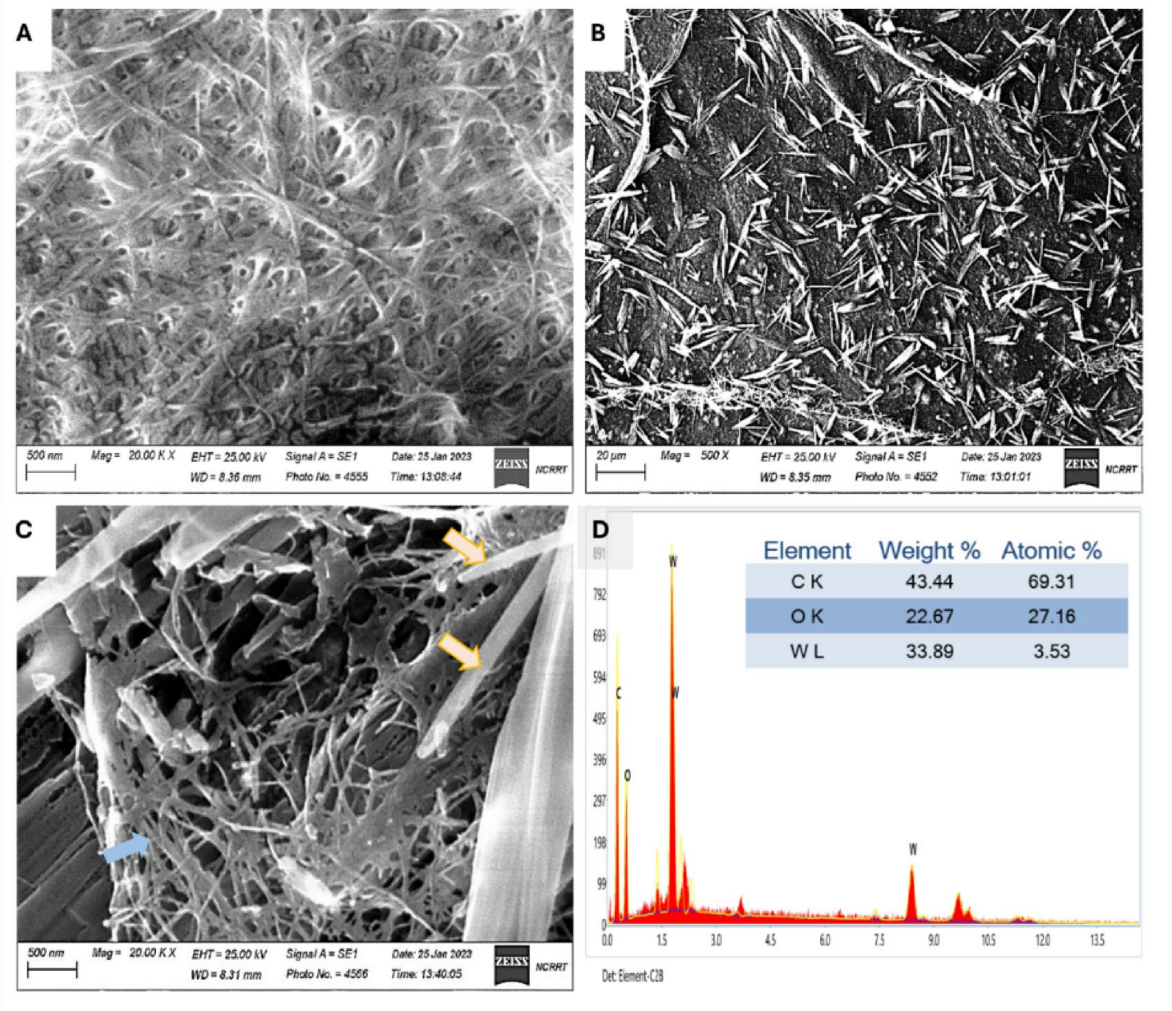
SEM of (**A**) the BC, (**B**) WO_3_ NWs, (**C**) BC/WO_3_ NWs: the blue arrow points to the BC nanofibers, while the orange ones indicate the compositing WO_3_ NWs, and (**D**) EDX analysis of the BC/WO_3_ NWs sample.

Regarding the microstructure inspected by the Scanning electron microscope (SEM), BC appears in Fig. 3A with the natural 3D network of entangled nanofibers including porous system, while the WO_3_ NWs appear as needle-shaped aggregates of mean size about 15–25 μm (Fig. 3B). The imaging of the BC/WO_3_ NWs composite showed the successful compositing route as it appears as a highly blended mixture of the cellulose nanofibers and the tungsten nanowires (Fig. 3C). Moreover, Energy Dispersive X-ray Spectroscopy (EDX) analysis reveals in Fig. 3D a prominent proportion of tungsten element in the sample, comprising more than 33% of the estimated sample mass, and about 3.5% of the atoms included. Considering the atomic number (74) and the atomic mass (183.84 amu) of tungsten element, these results are reasonable for a composite including cellulose nanofibers and tungsten oxide.

**Fig. 4:**
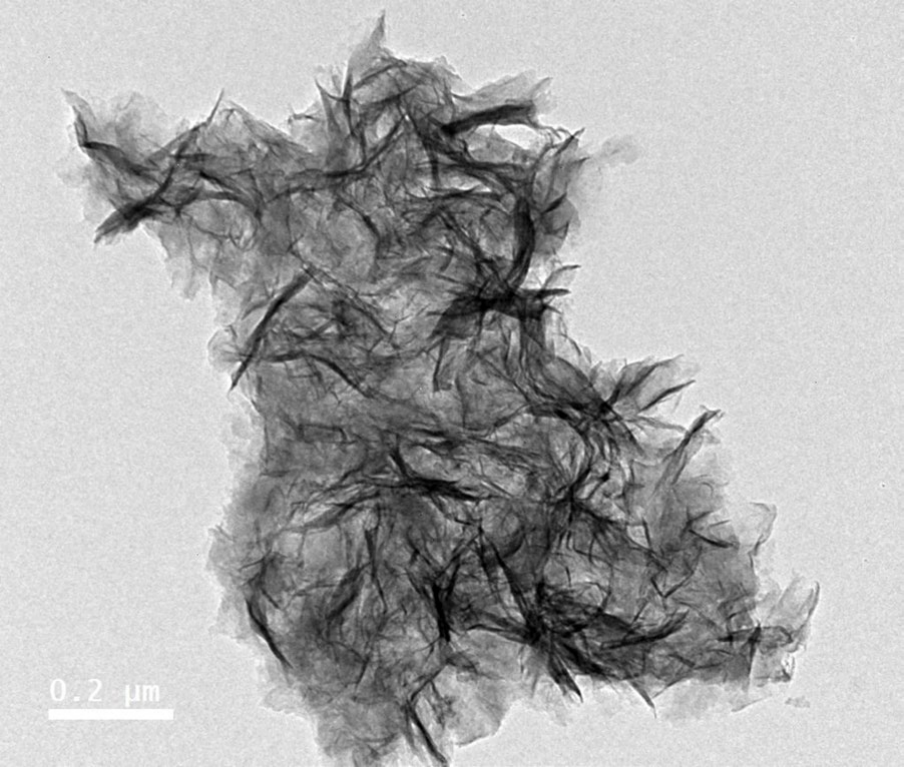
TEM imaging of WO_3_ NWs sample.

Transmission electronic microscope (TEM) imaging in Fig. 4 has revealed the needle shape of the WO_3_ NWs aggregates in clusters, where the size of these structures ranged between 200 and 400 nm. Unfortunately, TEM investigation for the BC/WO_3_ NWs sample had no outcomes since the nanofibrous nature of BC made the loading of the sample on the checking copper grid unattainable.

Regarding the observed discrepancy in WO₃ nanowire (NWs) dimensions between SEM and TEM imaging, we attribute this to the synthesis process, which likely produced tungsten nanowires with a broad size distribution. Each imaging technique has inherent limitations in magnification and resolution, affecting its ability to accurately capture specific size ranges. Moreover, in the case of BC/WO₃ NWs composite, SEM imaging tends to misidentify the nanoscale tungsten wires due to their morphological similarity to the cellulose nanofibers. Consequently, TEM analysis proved significantly more effective in distinguishing and characterizing the diverse size spectrum of the synthesized WO₃ nanowires.

**Fig. 5:**
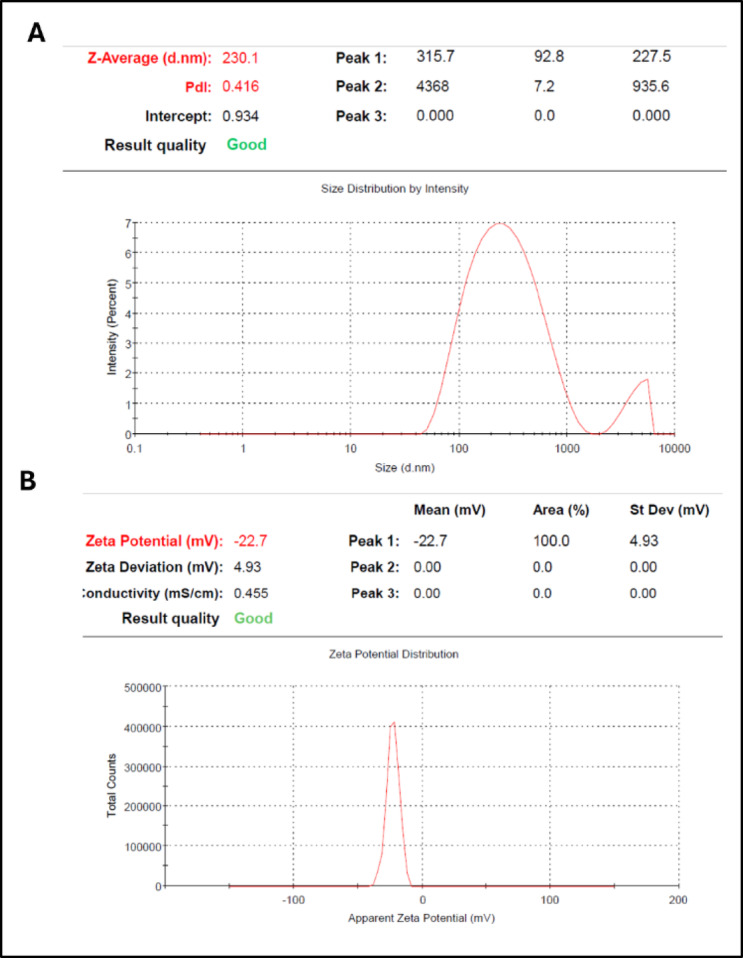
(**A**) Zeta potential, and (**B**) Zeta sizing of the WO_3_ NWs.

Figure 5A manifests the Zeta sizing analysis of the WO_3_NWs, where the measurement recorded average size 230.1 d.nm. Since the Zeta sizing technique is not fully effective with anisotropic nanoparticles, like the case of nanowires, we propose to present the results as attribution for the hydrodynamic diameter of the nanowires rather than the length^55,56^.

Zeta potential inspection has reported that the WO_3_ NWs are negatively charged with − 22.7 mV in water at room temperature (Fig. 5B). Tungsten nanoparticles have been ascribed with moderate stability in water at room temperature *via*recording − 12.9 mV^57^. This data is strongly linked to the water-based synthesis protocol employed for the composite, wherein the surface charges of the nanowires dispersed in the aqueous medium play a critical role in governing their interactions with the surrounding reactants.

**Fig. 6:**
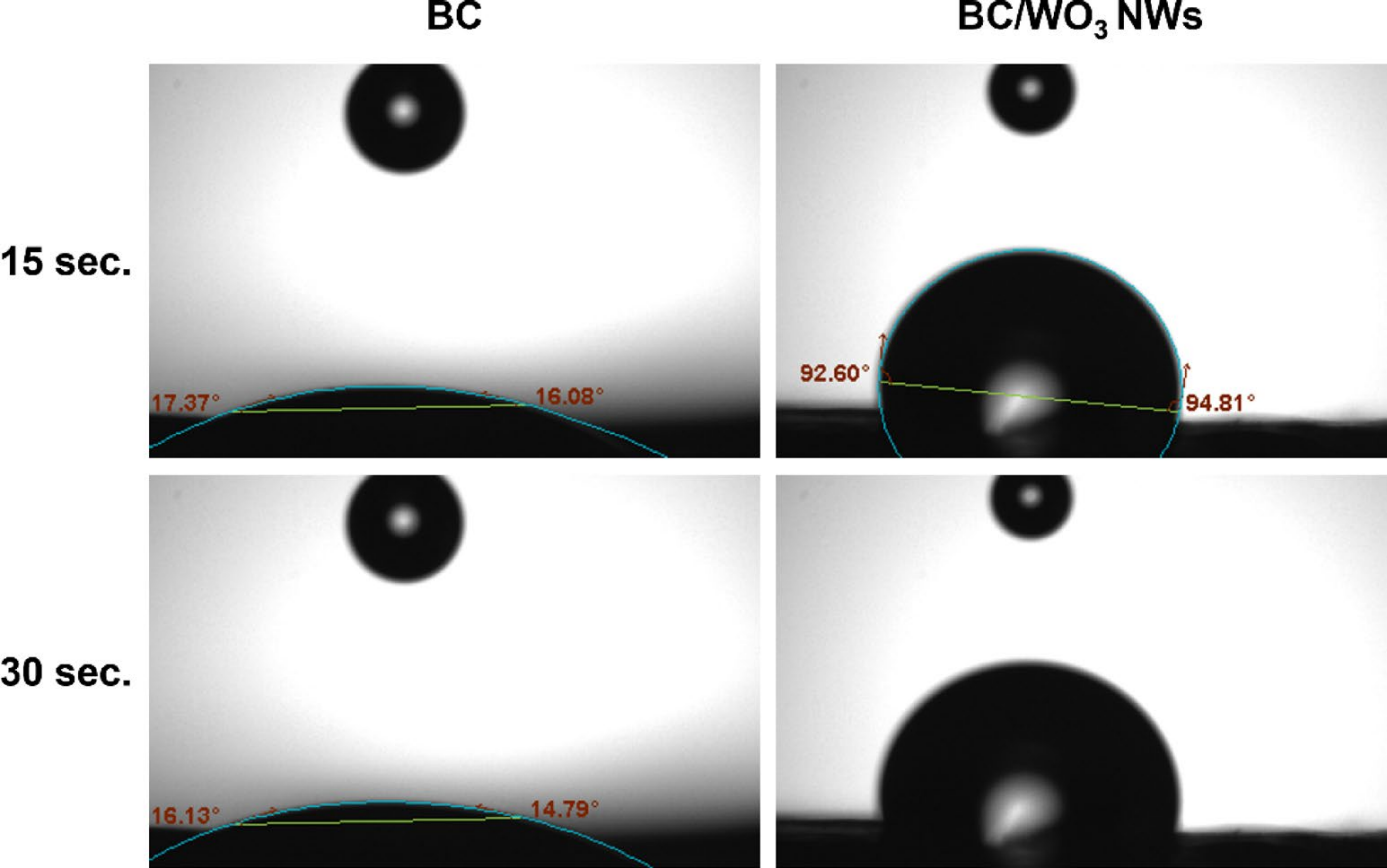
Contact angle results for the BC and BC/WO_3_ NWs composite.

Contact angle analysis was accomplished for the BC and BC/WO_3_ NWs composite to figure out the impact of WO_3_ NWs on the hydrophilicity of the BC nature. The examination elucidated that the composite exerted a prominent hydrophobicity comparing to the BC biopolymer, where the BC showed average contact angle of 20.3° and 15.5° at 15 and 30 s, respectively. Whilst, the BC/WO_3_ NWs composite recorded 94° and 96° at 15 and 30 s, in respective order (Fig. 6).

Based on the observed Zeta potential and contact angle measurements, it is presumed that the oxygen atoms within the WO₃ nanowire structure, together with the abundant hydroxyl groups distributed along the BC nanofibers, facilitate the formation of intermolecular O–OH hydrogen bonds during the compositing process. This interaction likely reduces the overall hydrophilicity typically associated with free hydroxyl groups, thereby contributing to a more stable and cohesive nanocomposite architecture.

**Fig. 7:**
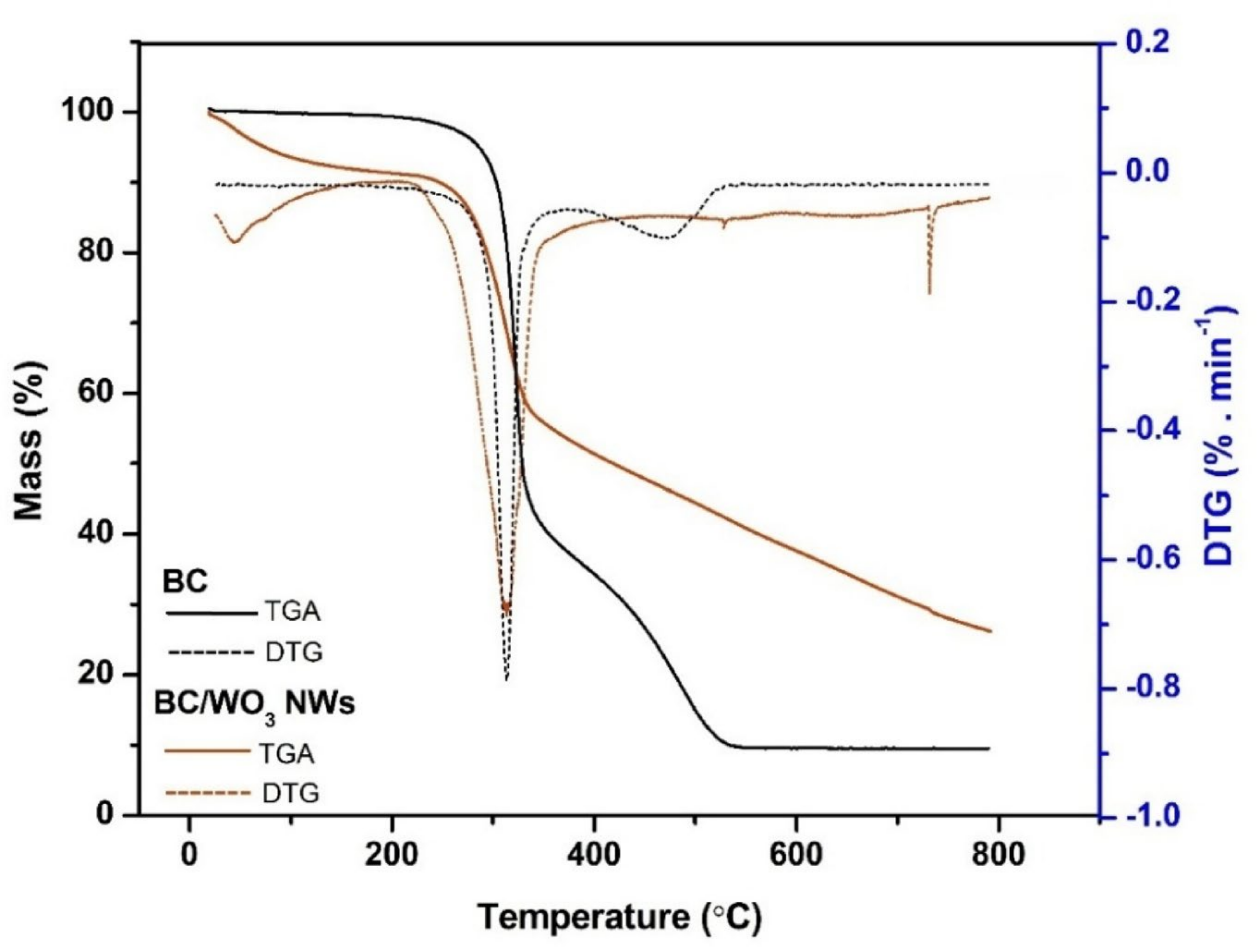
Thermal deterioration profile of the BC and BC/WO_3_ NWs composite including thermogravimetric analysis (TGA) and differential thermogravimetric analysis (DTG).

Thermogravimetric analysis (TGA) and differential thermogravimetric analysis (DTG) manifested dual-step and three-step deterioration for the BC and BC/WO_3_ NWs composite, respectively (Fig. 7). The BC phase transition exhibited thermostability till 328 °C before the decomposition takes place on two phases. On the other hand, the BC/WO_3_NWs composite lost about 7% of its mass by elevating the temperature to about 110 °C, which was attributed to the evaporation of the moisture content typically reported for BC^50^. Moreover, the stability of the composite was steady until the temperature 247 °C, recording 90% of its original mass. Beyond that temperature, the decomposition was sharp, where the mass plummeted to reach 56% at 348 °C and correlated with the deterioration of the included BC organic matrix. Afterward, the decomposition was steady, where the mass percent has decreased in the 349–791 °C temperature zone about 30% of the original mass. Chandrasekaran et al. studied the tungsten nanoflakes synthesized by phytoconstituents, where they attributed similar thermal behavior for the crystalline structure of the WO_3_nano-structures^58^.

The results indicate the potential stability of the composite in any application where the ambience temperature < 240 °C.

### Evaluation of the gamma attenuation of the BC/WO_3_ NWs composite

Several studies have analyzed the impact of various experimental conditions on the measured attenuation coefficient. Demir studied the effect of narrow/pencil beam geometries as well as the source-sample distance, utilizing H_2_O, PbO, and cellulose samples^59^. Another study investigated the quantitative analysis of detector collimator diameter effect, and attributed the deviation in measurements to the detection of elastic and inelastic scattered photons from the absorber^60^. Others explored the influence of sample thickness, underlining the contribution of multiple scattered photons as well as small-angle scattering photons^35,36,61^. On the other hand, Kurudirek and Medhat proposed an alternative approach to measure the normalized mass attenuation coefficients of materials with unknown thickness and density^62^.

**Fig. 8:**
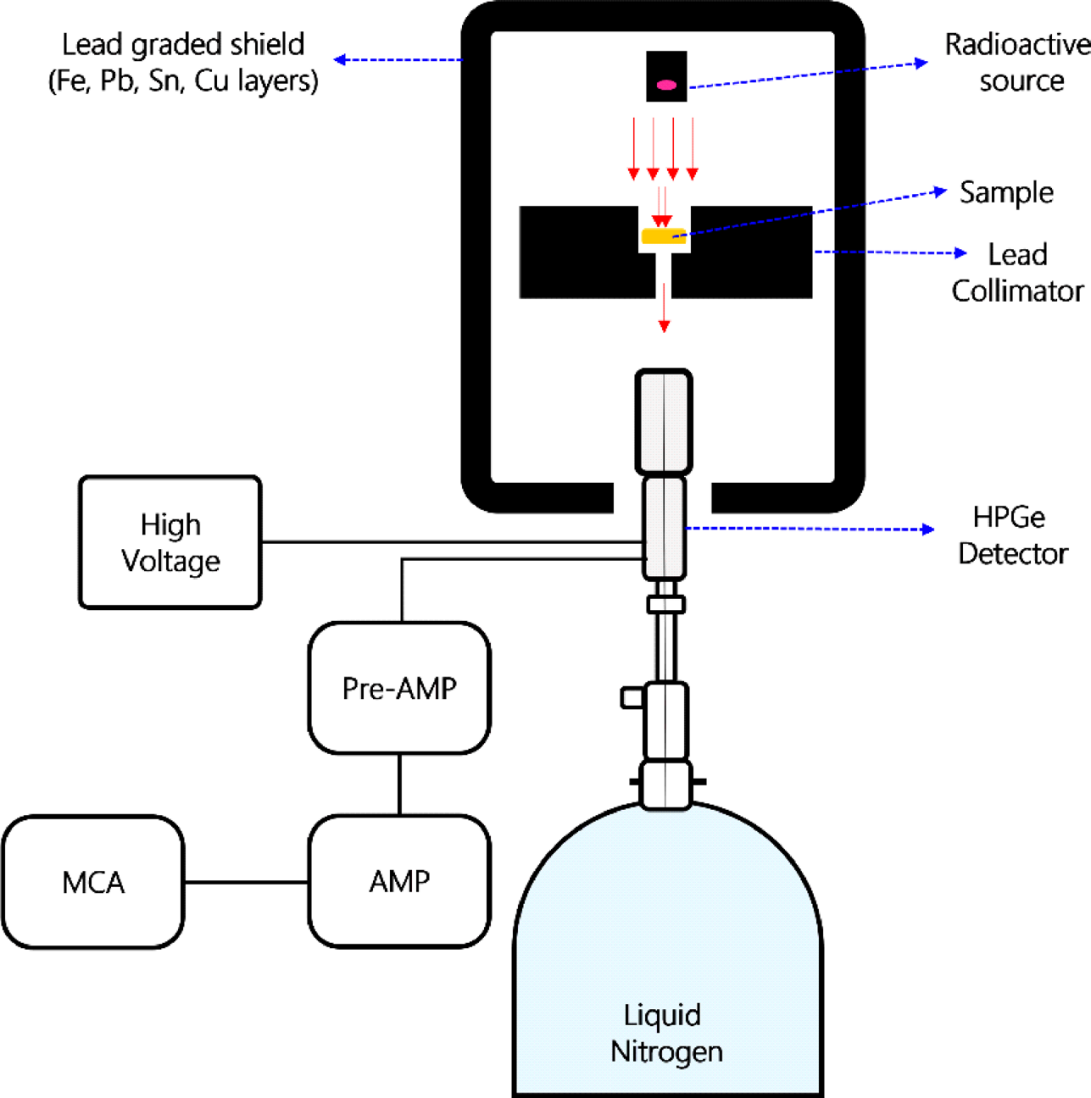
Schematic diagram for the photon measurement system.

In the current study, we adopted the “narrow beam” setup. A schematic diagram for the photon measurement system utilized for examination of BC/WO_3_ NWs shielding properties is pinpointed in Fig. 8. It consists of gamma spectrometer based on HPGe detector, the 10 cm lead multi-layers graded shield, the lead collimator and sample positioning system.

For studying the mass attenuation of the BC/WO_3_ NWs composite for gamma radiation, four discs of the BC/WO_3_ NWs were created with ascending WO_3_ NWs (wt%) to figure out the radioprotection capacity of the composite *versus* tungsten oxide content, as elucidated in Table 3.

**Table 3:** The specifications of the BC/WO_3_ NWs discs for mass attenuation measurement.

Sample code	Weight (g)	Thickness (mm)	Density (g/cm^3^)	WO_3_ (mg)	WO_3_ (wt%)
Cellulose + 0.3% WO_3_	1.66 ± 0.001	8.00 ± 0.05	1.56 ± 0.02	5 ± 1	0.30 ± 0.06
Cellulose + 1.1% WO_3_	1.20 ± 0.001	5.65 ± 0.05	1.61 ± 0.02	13 ± 1	1.08 ± 0.08
Cellulose + 6.8% WO_3_	1.17 ± 0.001	5.35 ± 0.05	1.66 ± 0.02	80 ± 1	6.81 ± 0.09
Cellulose + 11.7% WO_3_	2.13 ± 0.001	9.55 ± 0.05	1.69 ± 0.02	250 ± 1	11.69 ± 0.05

Long ago, X-ray attenuation by cellulose attracted the attention of investigators. For instance, Hoag and Krahmer investigated the attenuation of polychromatic X-ray energy through cellulose acetate for wood densitometry applications^63^. Recently, Kołodziejczyk et al. have examined the shielding properties of kombucha’s BC against gamma and neutron fields^24^. In addition, they tested metallic components (K, Fe, Mxenes) and biological additives for enhancing the radioprotection of kombucha’s bio-film. They concluded that BC is a possible candidate in a circular economy for future bioregenerative systems.

**Fig. 9:**
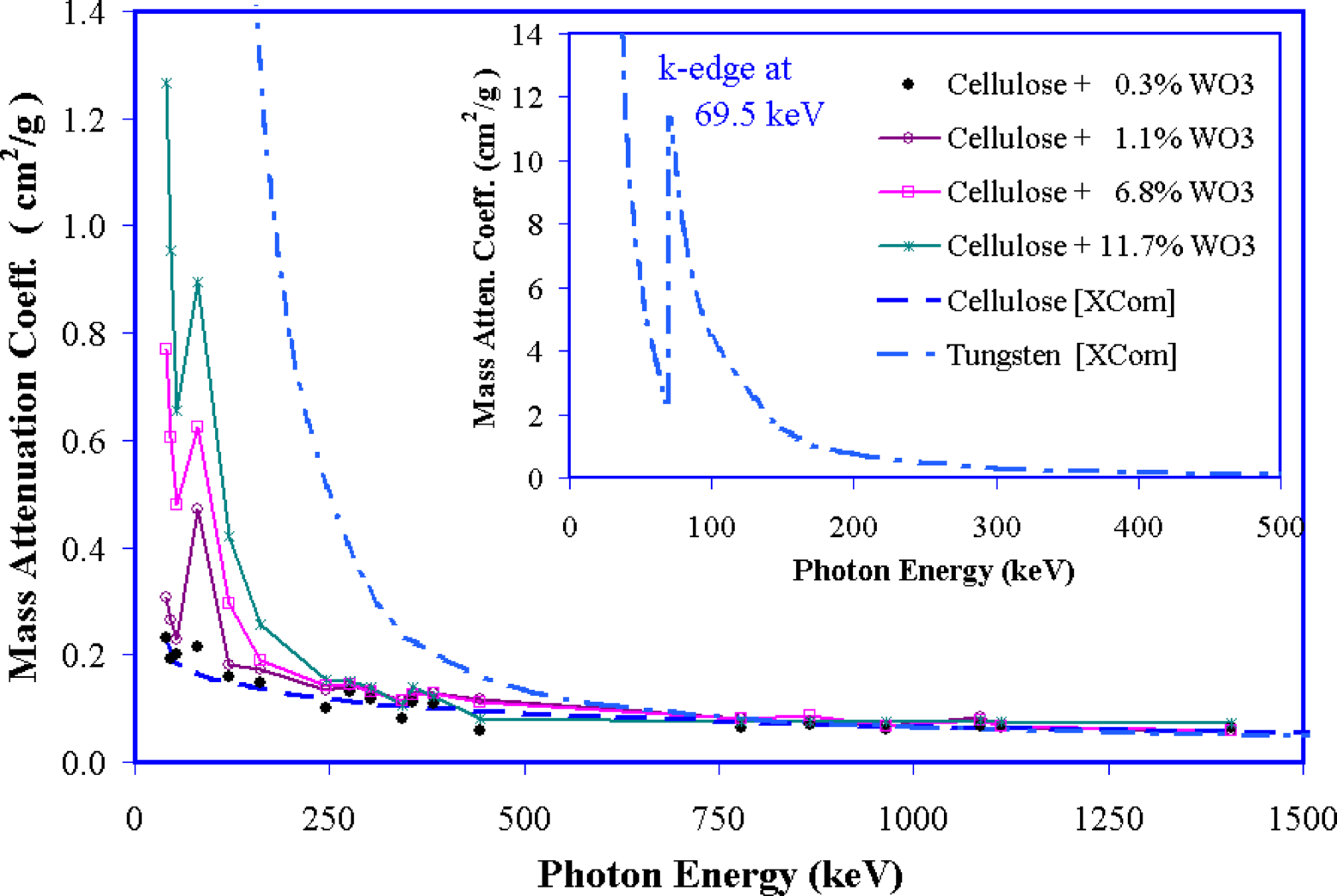
Measured mass attenuation coefficients as a function of incident photon energy for bacterial cellulose samples doped with tungsten oxide nanowires (WO_3_ NWs).

Figure 9; Table 4 show the mass attenuation coefficient of the composite of bacterial cellulose/tungsten oxide nanowires as a function of gamma energy for different concentration of.

WO_3_ NWs. For comparison, Fig. 9 additionally includes the data of pure cellulose as well as pure tungsten as calculated using XCOM code^64,65^. As expected, increasing the doping percentage enhances the shielding effectiveness of the measured samples. The only exception is for 0.3% (wt%) WO_3_ NWs, where attenuation coefficient is indistinguishable from pure BC sample within the precision limit of current measurements.

It is worth noting that the peak at ~ 81 keV of Ba-133 manifests the attenuation behavior at K-absorption edge of the tungsten at 69.52 keV. The K-edge corresponds to the abrupt increase in the photoelectric absorption of gamma radiation photons with energy just above the binding energy of the k-shell electrons.

**Table 4:** Measured mass attenuation coefficients (cm^2^/g) for the BC/WO_3_ NWs composites with different WO_3_ NWs content.

Energy (keV)	Mass attenuation coefficient (cm^2^/g)			
	BC + WO_3_ (0.3%)	BC + WO_3_ (1.1%)	BC + WO_3_ (6.8%)	BC + WO_3_ (11.7%)
39.90	0.23 ± 0.01	0.31± 0.00	0.77 ± 0.02	1.27 ± 0.03
45.58	0.19 ± 0.02	0.26± 0.01	0.61 ± 0.02	0.96 ± 0.03
53.16	0.20 ± 0.02	0.23± 0.02	0.48 ± 0.04	0.66 ± 0.03
81.00	0.21 ± 0.00	0.47± 0.01	0.63 ± 0.01	0.90 ± 0.01
121.78	0.16 ± 0.00	0.18± 0.00	0.30 ± 0.01	0.42 ± 0.01
160.61	0.15 ± 0.02	0.17± 0.03	0.19 ± 0.04	0.26 ± 0.02
244.69	0.10 ± 0.00	0.14± 0.00	0.14 ± 0.01	0.15 ± 0.00
276.39	0.13 ± 0.00	0.14± 0.00	0.15 ± 0.01	0.15 ± 0.00
302.85	0.12 ± 0.00	0.13± 0.00	0.13 ± 0.00	0.14 ± 0.00
344.27	0.08 ± 0.00	0.11± 0.00	0.12 ± 0.00	0.11 ± 0.00
356.01	0.11 ± 0.00	0.13± 0.00	0.13 ± 0.00	0.14 ± 0.00
383.84	0.11 ± 0.00	0.13± 0.00	0.13 ± 0.00	0.12 ± 0.00
443.96	0.06 ± 0.01	0.12± 0.01	0.11 ± 0.01	0.08 ± 0.01
778.90	0.07 ± 0.00	0.08± 0.00	0.08 ± 0.01	0.08 ± 0.00
867.38	0.07 ± 0.01	7.50E-2 ± 1.07E-2	0.09 ± 0.01	0.07 ± 0.01
964.05	0.06 ± 0.00	6.65E-2 ± 4.31E-3	0.07 ± 0.01	0.08 ± 0.00
1085.83	0.07 ± 0.00	8.42E-2 ± 5.13E-3	0.08 ± 0.01	0.08 ± 0.00
1112.07	0.06 ± 0.00	6.42E-2 ± 4.79E-3	0.07 ± 0.01	0.08 ± 0.00
1408.01	0.06 ± 0.00	5.97E-2 ± 3.59E-3	0.06 ± 0.00	0.07 ± 0.00

**Fig. 10:**
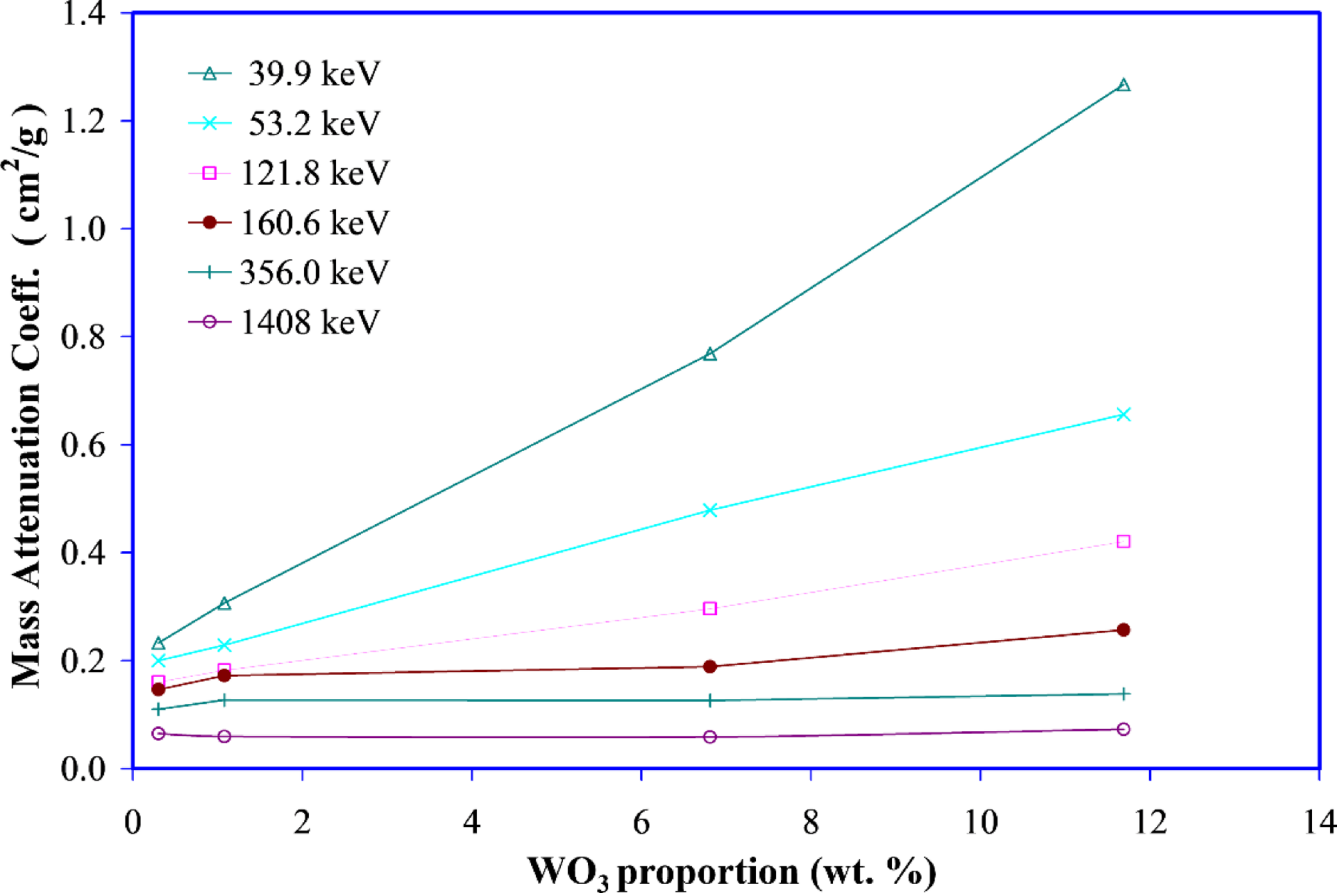
Measured mass attenuation coefficients as a function tungsten oxide nanowires (WO_3_ NWs) content of the synthesized composite.

Increasing the WO_3_ NWs content in the samples increases their mass-attenuation coefficient steeply at low energies (Fig. 10). This effect diminished with increasing the photon energy, whereas the photon energies above ~ 250 keV were correlated with almost no obvious shielding effect of nano-tungsten oxide doping regardless of the dopant concentration.

**Fig. 11:**
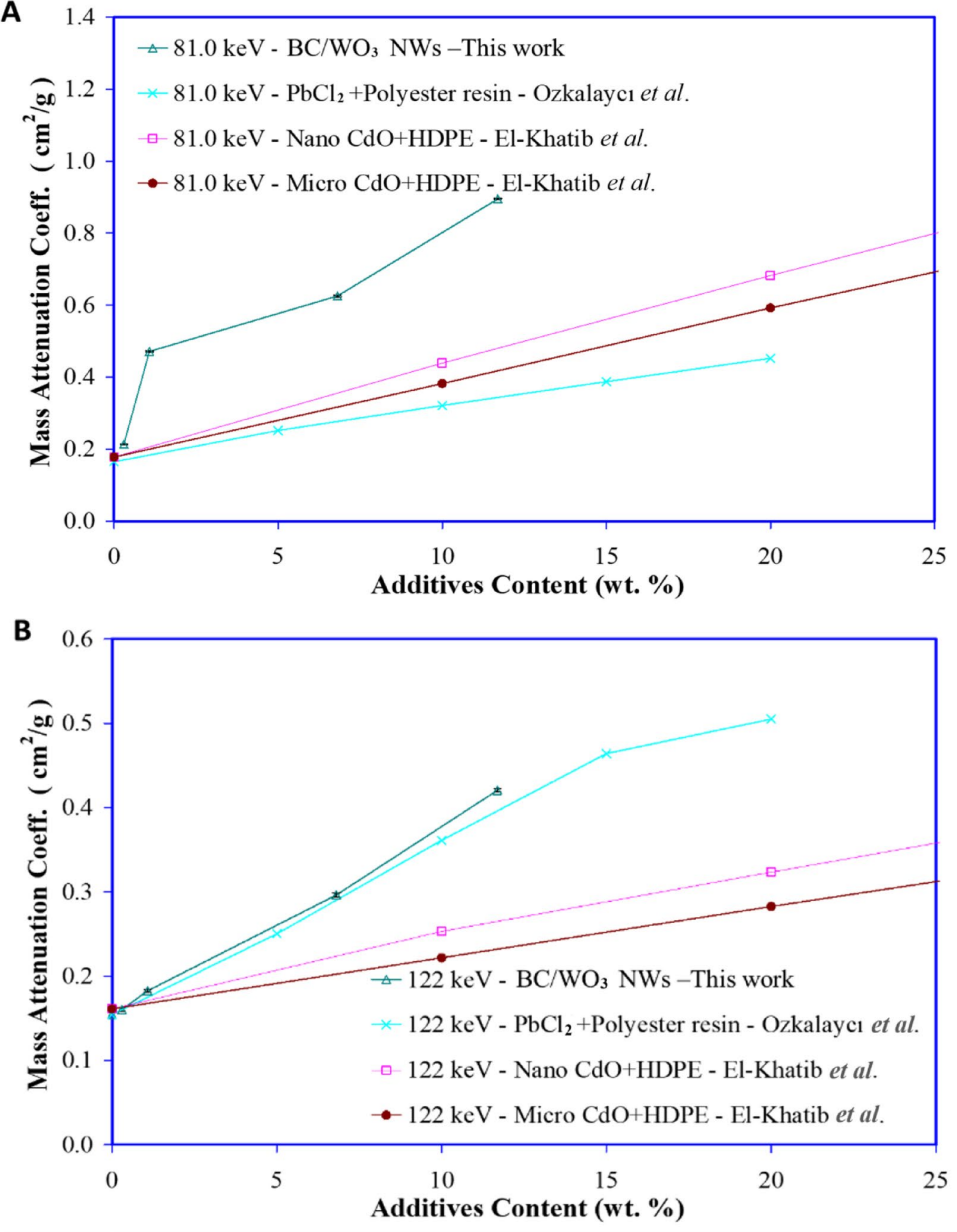
Measured mass attenuation coefficients as a function of metals content for different composites at photon energies (**A**) 81 keV and (**B**) 122 keV.

Comparing the BC/WO_3_ NWs composite with polyester-resin/PbCl_2_ and HDPE/CdO composites (Fig. 11) reveals that BC/WO_3_ NWs composite has a better shielding ability than others that based on PbCl_2_ and CdO only at energy as low as 81 keV (Fig. 11A)^5,66^, Thereafter, as the energy increases, cellulose/WO_3_ losses its superiority and its shielding capabilities becomes comparable to the polyester-resin/PbCl_2_, and both are better than the HDPE/CdO composites (Fig. 11B).

**Fig. 12:**
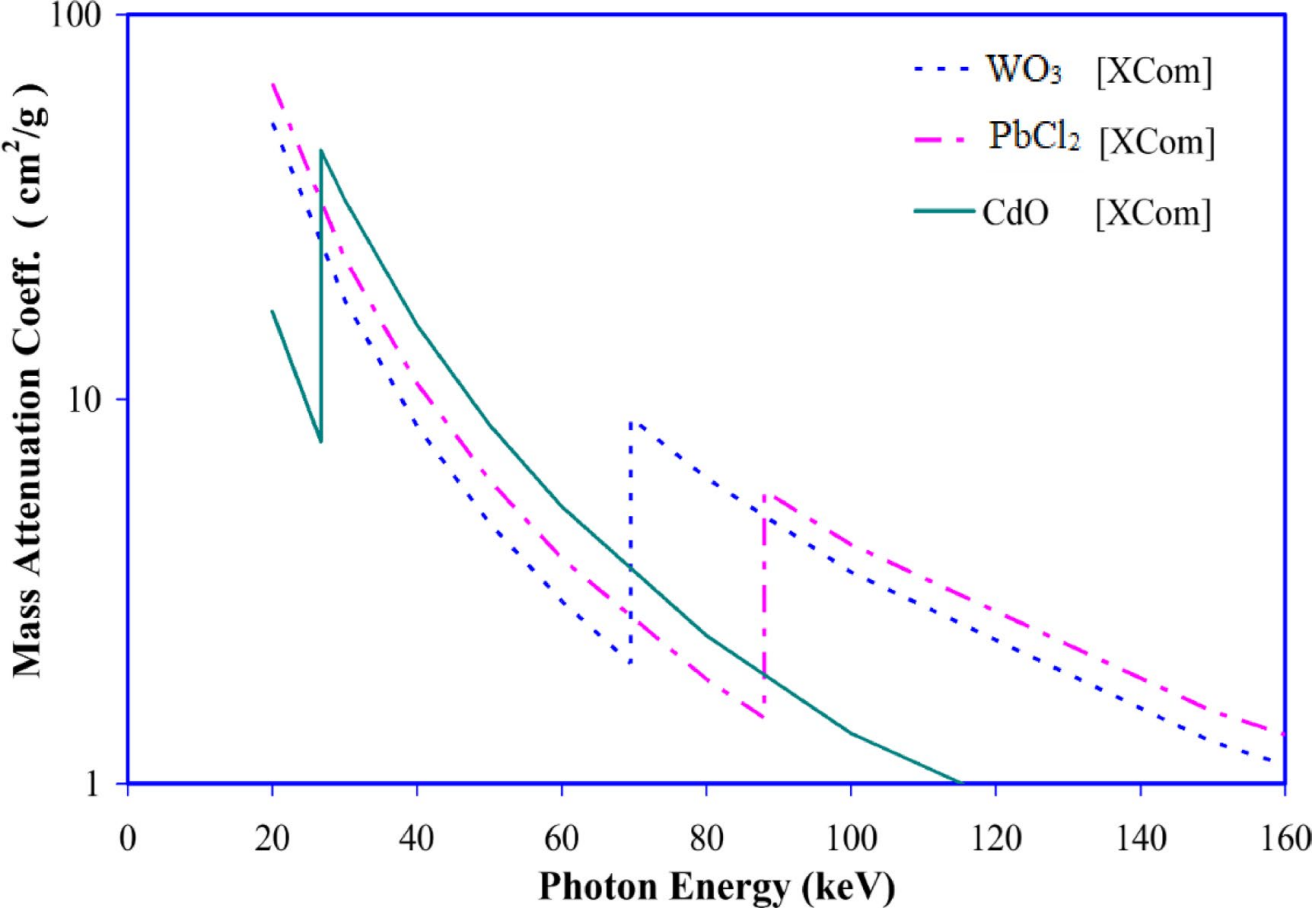
The calculated mass attenuation coefficients of WO_3_ comparing to PbCl_2_ and CdO at ascending photon energy values (keV).

Above observations may be explained in the light of the photoelectric interaction of W, Pb, and Cd. Although, the photoelectric interaction probability increases with increasing the material atomic number, however, it shows exceptional sharp rise at energies just above the binding energy of the k-shell electrons, thus called K-absorption edge. The k-edge energies of Cd, W and Pb are 26.71, 69.53, 88.0 keV respectively. Consequently, each element has its own window of superiority to expose its shielding power. Hence, as inferred from Fig. 12, Cd based compounds have the highest shielding ability between energies 26.71–69.53 keV, while W based compounds have the strongest shielding potential between 69.53 and 88.0 keV, whereas the Pb compounds cover other energy regions outside these energy windows owing to the Pb high atomic number.

In summary, the BC/WO₃ NWs composite offers a promising lightweight and eco-friendly alternative to conventional radioprotective materials, effectively addressing the limitations of heavy metals and non-sustainable synthetic polymers. Its unique combination of structural integrity, reduced hydrophilicity, and gamma-ray attenuation potential positions it as a viable candidate for advanced shielding applications. To fully realize its industrial potential—particularly in medical devices and aerospace technologies—further refinements in composition and performance optimization are essential to meet stringent regulatory and functional standards.

## Conclusion

In pursuit of a sustainable alternative to conventional radiation shielding materials, we have developed a novel composite through an environmentally friendly synthesis route. This composite integrates bacterial cellulose (BC)—a biodegradable and biocompatible polymer—with varying concentrations of tungsten trioxide nanowires (WO₃ NWs), aiming to replace traditional shielding systems that rely on either dense intrinsic materials or synthetic, non-eco-friendly polymers.

The resulting BC/WO₃ NWs composite exhibited reduced hydrophilicity and demonstrated acceptable thermal stability, indicating its potential for structural integrity under operational conditions. Prior to evaluating its shielding performance, these physicochemical properties were assessed to ensure compatibility with radiation environments. Subsequent gamma-ray attenuation tests across a broad energy spectrum revealed a clear dependence on the WO₃ NWs loading. The BC/WO₃ NWs composite exhibits enhanced shielding performance at low photon energies, demonstrating clear superiority over other conventional systems. As the energy increases, its effectiveness becomes comparable to alternative composites, maintaining a competitive edge. These findings underscore the composite’s suitability for low-energy radiation shielding applications, with tunable performance based on nanowire concentration.

Future research directions will focus on optimizing the composition of this environmentally friendly composite to improve its gamma-ray attenuation capabilities, particularly at higher energy levels, where the current performance shows limitations. This will involve systematic modification of the nanowire loading, matrix architecture, and potential incorporation of synergistic additives to enhance shielding efficiency. Additionally, in-depth analysis of the interfacial bonding mechanisms between the bacterial cellulose matrix and the embedded WO₃ nanowires will be conducted. Understanding these molecular interactions will provide valuable insights into structural reinforcement strategies and guide the development of more robust, high-performance shielding materials tailored for advanced radiation environments.

## Data Availability

All data supporting the findings of this study are included within the paper.
